# Using Machine Learning to Predict ICU Transfer in Hospitalized COVID-19 Patients

**DOI:** 10.3390/jcm9061668

**Published:** 2020-06-01

**Authors:** Fu-Yuan Cheng, Himanshu Joshi, Pranai Tandon, Robert Freeman, David L Reich, Madhu Mazumdar, Roopa Kohli-Seth, Matthew A. Levin, Prem Timsina, Arash Kia

**Affiliations:** 1 Institute for Healthcare Delivery Science; Icahn School of Medicine at Mount Sinai, 1425 Madison Avenue, New York, NY 10029, USA; Fu-Yuan.Cheng@mountsinai.org (F.-Y.C.); Himanshu.Joshi@mountsinai.org (H.J.); Robert.Freeman@mountsinai.org (R.F.); Prem.Timsina@mountsinai.org (P.T.); Arash.Kia@mountsinai.org (A.K.); 2 Department of Population Health Science and Policy, Icahn School of Medicine at Mount Sinai, 1425 Madison Avenue, New York, NY 10029, USA; 3 Respiratory Institute, Icahn School of Medicine at Mount Sinai, 10 E 102nd St, New York, NY 10029, USA; Pranai.Tandon@mountsinai.org; 4 Hospital Administration; The Mount Sinai Hospital, 1 Gustave L. Levy Place, New York, NY 10029, USA; David.Reich@mountsinai.org; 5 Department of Anesthesiology, Perioperative and Pain Medicine, 1 Gustave L. Levy Place, Icahn School of Medicine at Mount Sinai, New York, NY 10029, USA; Matthew.Levin@mssm.edu; 6 Institute for Critical Care Medicine, Icahn School of Medicine at Mount Sinai, New York, NY 10029, USA; Roopa.Kohli-Seth@mountsinai.org; 7 Department of Genetics and Genomic Sciences, 1 Gustave L. Levy Place, Icahn School of Medicine at Mount Sinai, New York, NY 10029, USA

**Keywords:** COVID-19, critical care, supervised machine learning, random forest, intensive care units

## Abstract

Objectives: Approximately 20–30% of patients with COVID-19 require hospitalization, and 5–12% may require critical care in an intensive care unit (ICU). A rapid surge in cases of severe COVID-19 will lead to a corresponding surge in demand for ICU care. Because of constraints on resources, frontline healthcare workers may be unable to provide the frequent monitoring and assessment required for all patients at high risk of clinical deterioration. We developed a machine learning-based risk prioritization tool that predicts ICU transfer within 24 h, seeking to facilitate efficient use of care providers’ efforts and help hospitals plan their flow of operations. Methods: A retrospective cohort was comprised of non-ICU COVID-19 admissions at a large acute care health system between 26 February and 18 April 2020. Time series data, including vital signs, nursing assessments, laboratory data, and electrocardiograms, were used as input variables for training a random forest (RF) model. The cohort was randomly split (70:30) into training and test sets. The RF model was trained using 10-fold cross-validation on the training set, and its predictive performance on the test set was then evaluated. Results: The cohort consisted of 1987 unique patients diagnosed with COVID-19 and admitted to non-ICU units of the hospital. The median time to ICU transfer was 2.45 days from the time of admission. Compared to actual admissions, the tool had 72.8% (95% CI: 63.2–81.1%) sensitivity, 76.3% (95% CI: 74.7–77.9%) specificity, 76.2% (95% CI: 74.6–77.7%) accuracy, and 79.9% (95% CI: 75.2–84.6%) area under the receiver operating characteristics curve. Conclusions: A ML-based prediction model can be used as a screening tool to identify patients at risk of imminent ICU transfer within 24 h. This tool could improve the management of hospital resources and patient-throughput planning, thus delivering more effective care to patients hospitalized with COVID-19.

## 1. Introduction

With more than 3 million cases and 200,000 deaths [1] by the end of April 2020, the COVID-19 pandemic has rapidly emerged as a serious global health emergency [2], testing the ability of health care systems to respond. The burden on health care systems emanates both from the high incidence of COVID-19 and the fact that 20% to 30% of patients experience a moderate-to-severe form of the disease—with multi-organ failure, prolonged periods of morbidity and hospitalization, and high mortality [3]. Moreover, from 5% to 12% of all patients diagnosed with COVID-19 and up to 33% of hospitalized patients require supportive critical care in an intensive care unit (ICU) [3,4,5]. These estimates indicate that the rate of ICU transfer of hospitalized patients with COVID-19 is significantly higher than the ICU transfer rates of 11% reported for other hospitalized patients [6,7].

Furthermore, the need for ICU care may be even higher in specific high-risk groups with COVID-19, such as older individuals [3] or those with pre-existing comorbidities [8]. For example, over 75% of COVID-19 patients admitted to the ICU have one or more pre-existing comorbid conditions [9]. According to an estimate by the American Hospital Association, there are just under 100,000 ICU beds in the United States [10], with over 67% occupancy under normal circumstances [11]—a potential constraint on resources during a surge in cases. Moreover, constraints in the availability of trained manpower [12] may occur with a rapid surge in COVID-19 hospitalizations. COVID-19 patients admitted to non-ICU units often experience rapid clinical deterioration [13] and, therefore, require frequent clinical assessments. However, with resources stretched thin, frequent assessment is difficult and can increase the risk of exposure among frontline personnel. To efficiently manage these finite resources and personnel, optimal prioritization of patients and efficient use of hospital resources are necessary.

ICU care may be needed for supportive management of severe COVID-19-associated pneumonia, acute respiratory distress (ARDS), sepsis, cardiomyopathy, arrhythmia, and acute renal failure. ICU care also may become necessary to manage prolonged hospitalization-associated complications, such as coagulopathy [14], secondary infections, gastrointestinal bleeding, and other problems [13]. Determining whether an individual’s dynamic risk of clinical deterioration warrants an ICU transfer may require analyses of temporal changes in patients’ conditions and key indicators of imminent complications of COVID-19. Supervised machine learning approaches may be useful to (a) analyze and interpret patients’ clinical and laboratory values and their temporal changes, and (b) quantify their dynamic risk of clinical deterioration and the need for ICU transfer.

The primary aim of this study is to develop a novel supervised machine learning classifier for predicting the risk of ICU transfer within the next 24 h for COVID-19 patients using hospital EMR data. We applied a random forest (RF) [15] approach, which has proven promising in analyzing complex clinical data of multiple types [15], has high model generalizability [15], and can elucidate high-order interactions between variables without compromising predictive accuracy [16]. We describe the development and validation of such a model, its predictive performance, and the interpretation of our results.

## 2. Materials and Methods

### 2.1. Study Cohort and Features

This study was approved by the Mount Sinai Health System Institutional Research Board (IRB protocol number: 18-00581); the need for informed consent was waived.

The study cohort was comprised of patients 18 years or older who had a COVID-19 diagnosis and were admitted to the Mount Sinai Hospital in non-ICU general in-patient beds between 26 February and 18 April 2020. The diagnosis was based on a clinical conclusion of an infectious disease specialist or a positive PCR test (initial or repeat testing).

The following data were retrospectively collected from the Mount Sinai Health System COVID-19 registry, sourced from an EPIC EHR system: demographic information, time-series of the admission–discharge–transfer events, structured and semi-structured clinical assessments, vital signs from nursing flowsheets, and laboratory and electrocardiogram (ECG) results.

### 2.2. Sampling Strategy

Given the crisis nature of the pandemic, clinicians caring for this cohort collected data such as vital signs, diagnostic labs, ECGs, and nursing assessments based on clinical judgment and resource availability rather than a standard protocol. Thus, to create time-series data for each observational variable, we included the three most recent assessments available when the feature vector was created. Feature vectors were created daily during each COVID-19 patient’s non-ICU general bed stay until discharge, ICU transfer, or death. Missing values for each variable were imputed by using the median value across the cohort [17].

### 2.3. Labeling

**Figure 1:**
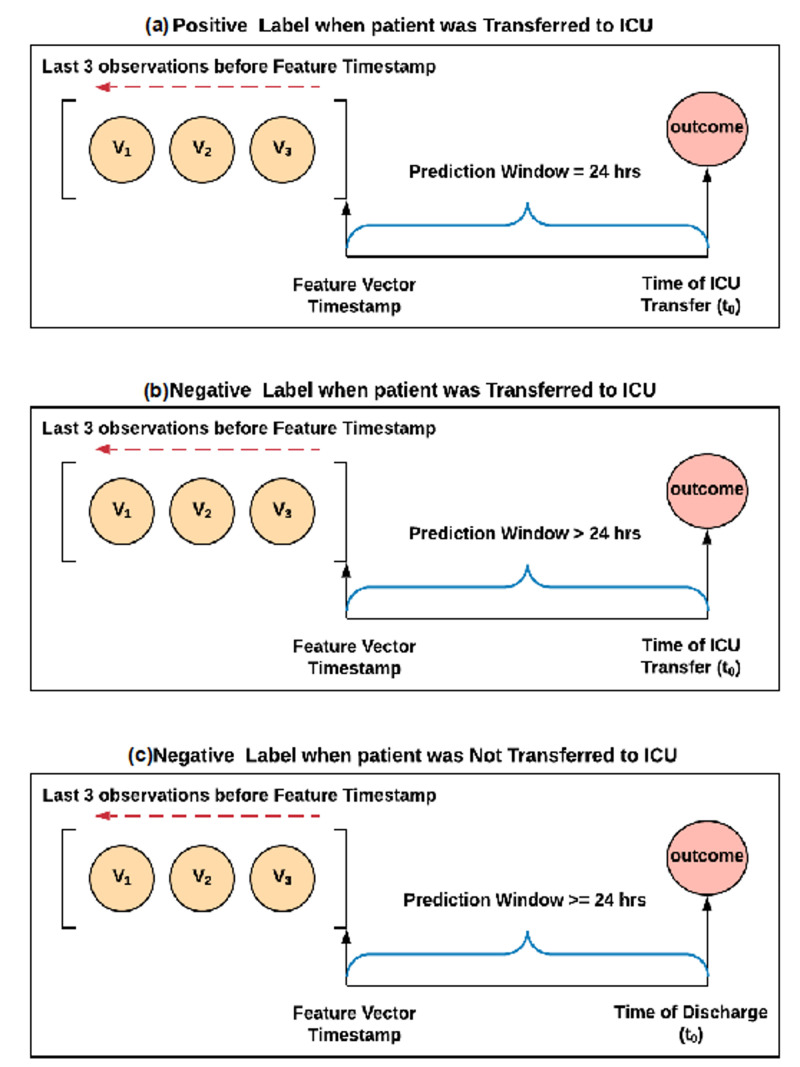
Feature vector labeling strategy. (**a**) Basis for positive labels; (**b**) and (**c**) basis for negative labels. V_1–3_: observations used for creating the feature vector; t_0_: time of ICU transfer.

The primary outcome of this study was ICU transfer within 24 h from the time of prediction. Labeling of feature vectors followed the following logic: (1) If the ICU transfer was within 24 h of the feature vector creation, we labeled the feature vector as positive; (2) If the ICU transfer occurred after 24 h from the creation of the feature vector, we labeled the feature vector as negative; (3) If the ICU transfer did not occur during the patients’ stay, then all feature vectors for that admission were labeled as negative. This process is depicted in Figure 1.

### 2.4. Training, Testing, and Cross-Validation

The study cohort data were randomly split into a training set used for training the prediction model, and a test set used for testing the model’s performance. The training set consisted of 70 percent of the full cohort, and the test cohort consisted of the remaining 30 percent. We randomly split our cohort so that patients were only included in the training or the test set. The non-ICU bed to ICU transfer rate in our cohort was 3.7 percent, which created an extreme class imbalance between the majority class (feature vectors without the occurrence of ICU transfer within 24 h) and the minority class (feature vectors with ICU transfer within 24 h). We performed random under-sampling [18,19] on the training data set for balancing the majority class (negative label) until both classes were equally balanced.

The RF model was trained with 10-fold cross-validation. The open-source Apache Spark project machine-learning library [20] was used.

### 2.5. Feature Selection

The features included in this study were based on clinical judgments and reports in the COVID-19 literature. We included periodic monitoring of vital signs [21], complete blood count, serum biochemical tests [22], coagulation profile [14], and electrocardiogram results [23] as relevant input variables. The full list of features used in modeling is provided in Table S1. Features were ranked by using the Gini importance [20].

### 2.6. Model Testing

The model performance was evaluated on the test set. RF model-derived class probabilities [20] were used to predict ICU transfer within 24 h with a default threshold of ≥0.5. Predictions less than the default threshold were categorized as negative. Sensitivity, specificity, accuracy, and area under the receiver operating curve (AUC-ROC), along with 95% CI, were estimated for evaluating the screening tool’s performance [24]. Performance metrics were computed in the R environment [25] by using custom scripts and R packages—PRROC (v.1.3.1) [26], pROC (v. 1.15) [27], and epiR (v. 1.0.4) [28].

## 3. Results

### 3.1. Cohort Characteristics

Cohort characteristics are shown in Table 1. The study cohort yielded 9639 feature vectors, which contained data from each day of non-ICU hospital stay for 1987 unique patients. Each individual vector, generated 24 h apart, represented a day of in-patient stay in a non-ICU bed for each patient. The split cohort resulted in 5548 and 2386 feature vectors created from the stays of 1168 and 521 patients in the training and test datasets, respectively. After performing majority-class under-sampling, the final training set consisted of 2008 feature vectors, representing each non-ICU stay of 401 unique patients. The median time to ICU transfer from the time of admission was 2.45 days.

The study cohort included a higher proportion of women, and about two-thirds of the cohort was between 18 and 65 years old. The median duration of hospital stay was 4.2 days and ranged between 1 to 43.6 days. About one-quarter of the patients in the cohort had more than one comorbidity, including COPD, diabetes, hypertension, obesity, or cancer.

### 3.2. Features and Model Hyperparameters

A total of 31 variables (comprising 99 features) had predictive value using the Gini importance metric in training the RF model. Hyper-parameters used in the final model are provided in Table S1.

### 3.3. Predictors and Their Importance

**Figure 2:**
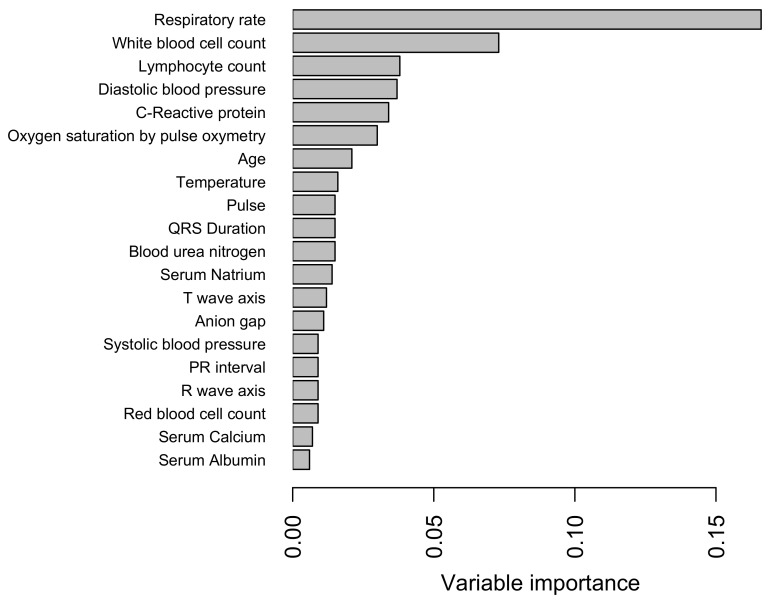
Gini importance: top 20 predictive variables.

The top 20 predictive variables are summarized in Figure 2. Model input variables with their respective sources are listed in Table S2. Our model identified a series of features related to progressive respiratory failure (respiratory rate, oxygen saturation), markers of systemic inflammation (C-reactive protein, white blood cell count), shock (systolic and diastolic blood pressures), renal failure (blood urea nitrogen, anion gap, and serum creatinine), and the pathophysiology of COVID-19 (lymphocyte count). Respiratory rate (the earliest recorded value of the latest three assessments) had the highest predictive value in the RF model, and white blood cell count was the second highest. Variables included in the final model reflected the importance of temporal changes in vital signs, markers of acid-base equilibrium and systemic inflammation, and predictors of myocardial injury and renal function.

### 3.4. Predictive Performance of the Model

**Figure 3:**
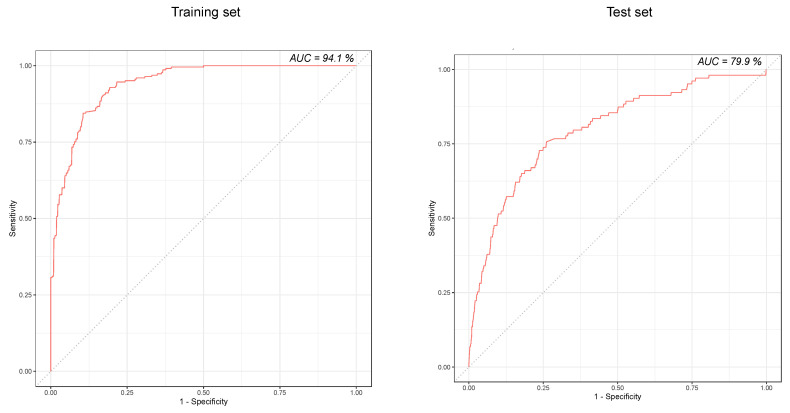
Receiver operating characteristic curve of the prediction model on training set (**left**) and test set (**right**).

The predictive performance of the RF-based model on the test dataset is presented in Table 2. Of 2386 feature vectors, 89 represented patient-days where ICU transfer occurred within 24 h of the prediction time point. The AUC-ROC of the prediction model is shown in Figure 3.

## 4. Discussion

Our model provides a tool for dynamic risk quantification for ICU transfer within the next 24 h. Clinical management of COVID-19 requires frequent monitoring and re-assessment among patients who may suffer rapid deterioration. Although deterioration may be evident by corroboration of changes in vital signs, laboratory results, electrocardiograms, and information in nursing notes, frequent review of these important parameters might not be feasible in crisis situations. Using machine learning, we developed a model for identifying deteriorating patients in need of ICU transfer by using data routinely collected during inpatient care. This model could be easily automated as an alternative to manual clinical review. Furthermore, inspection of important features in the model can provide insight into predictors and their plausible links to the pathophysiology of clinical deterioration among patients with COVID-19.

### 4.1. Model Variables of Interest

A key advantage of using an RF-based model is that the relative importance of predictive features is available for end users to interpret. Our finding that lymphocyte count is a significant predictor of ICU transfer correlates with previous reports that identified lymphopenia as a predictor of severe disease and poor prognosis [29,30].

Although age is clearly identified as a risk factor for needing ICU care among patients with COVID-19 [3], patients above 65 years old have lower rates of ICU transfer, despite higher mortality [5], possibly reflecting a greater preference for palliative or less aggressive care in older patients. We believe that the relatively low rank of age as a risk factor in our model could mean that our model incorporates actual patient data and patterns of clinical practice into its predictions.

Acute worsening of respiratory rate and oxygen saturation are used for identifying COVID-19 patients at risk of developing acute respiratory distress syndrome [31,32]. The model ranks oxygen saturation with a significantly lower predictive value than respiratory rate. A significant proportion of COVID-19 patients who are hospitalized need supplemental oxygen support. One possible explanation underlying the lower predictive value of oxygen saturation is that in patients with progressive hypoxia, a progressively greater fraction of inhaled oxygen (FiO_2_) is delivered to maintain adequate percutaneous oxygen saturation (SpO_2_) until the patient can no longer maintain normal oxygen saturation despite support from high-flow nasal oxygen or non-invasive ventilation. This makes SpO_2_ a less sensitive reflection of disease progression until severe respiratory decompensation occurs. We propose to include FiO_2_, level of respiratory support, and SpO_2_ as variables in future versions of this model.

C-reactive protein has been reported as a marker of disease severity in early phases of COVID-19 infection and is positively correlated with COVID-19 pneumonia [33]. Patients’ vital signs (e.g., pulse rate, blood pressure, and temperature) are among the top 20 predictors in this model and are widely accepted as identifying patients in critical condition who are at risk of deterioration [34]. Hematologic parameters such as red blood cell count, hemoglobin, platelet count, and white blood cell count are conventionally used markers of sepsis in critical care settings [35]; thus, it is not surprising that they were predictive of COVID-19 in our model also. Abnormalities in potassium, sodium, and calcium also have been associated with severe COVID-19 [36].

### 4.2. Model Strengths

Our model has strengths in terms of methodology, utility, and scalability. The labeling approach of feature vectors—using the last 3 observations, rather than the earliest or latest—made it easier to minimize chances of over-fitting despite the low sample size for training. The cohort is diverse in distribution of key variables such as age, race, ethnicity, and length of hospitalization, supporting the generalizability of the model. The model uses input variables mainly comprised of routine laboratory and clinical data, which are commonly available in most streaming data models across the U.S. Furthermore, the model can be adopted to different frequencies of assessments and different common input variables. It can be adjusted to use streaming data from the EMR and provide frequent predictions for real-time risk prioritization. We use the Fast Healthcare Interoperability Resources (FHIR) format for facilitating data exchange and retrieval from an EPIC-supported EMR system. This can help to improve the model’s scalability in other hospital settings.

Clinical judgment and resources can play a significant role in data availability. In addition, clinical documentation may not be perfect during crises, when normal documentation standards are relaxed due to the high work burden of clinicians. Therefore, unavailable data (as in our case) may be the consequence of either clinical judgement on need for specific assessments or imperfect clinical documentation.

Despite the non-random pattern of data availability for specific variables, the imputation strategy and the RF model had reasonably high sensitivity. This supports previous reports that found RF models to be highly suitable in situations with missing data [17], complex non-linear relationships among input variables, and their potential higher-level interactions [16]; thus, an ensemble-based classification approach minimizes overfitting [15]. An additional asset of this model is that, unlike other models, key discriminatory variables underlying each prediction can be provided.

### 4.3. Model Limitations

Low sample size and class imbalance resulting from low ICU transfer rates are major limitations to this version of the model, which resulted in low precision. Therefore, we recommend using this version of the model as a prioritization tool, not a tool for clinical decision support. Since the model is based on data from a single hospital, its case mix may not be easily generalizable to other settings. For example, in this cohort, rates of hypertension and diabetes were lower than in others reported [4,5,37]. Variables related to systemic inflammation and the coagulation cascade (e.g., D-dimer, fibrinogen, ferritin, and lactate dehydrogenase) were not available for modeling when this model was generated. While our model provides high sensitivity, we believe that inclusion of these other markers, which have predictive and/or prognostic value [38], could improve subsequent iterations of the model.

While SpO_2_ without assessments of FiO_2_ and level of oxygen support may not be sufficient to capture signs of progressive hypoxia, the inclusion of all three variables in subsequent versions of the model could also further improve its performance. However, given the low sample size of a single medical center in the acute phase of a pandemic, it may be difficult to generate a model with both high sensitivity and precision (positive predictive value).

### 4.4. Practice Implications

As a screening tool for development of critical illness, this model has multiple opportunities for clinical use. In addition to identifying patients with a potentially increased need for ICU transfer within 24 h, the tool can also be used for improving the coordination of patient transfers to the ICU. The tool can be used to inform clinicians of patients at higher risk of a greater need for frequent assessments, and thereby can facilitate inclusion of clinicians less familiar with critical care medicine.

Earlier identification of high-risk patients could potentially reduce the use of invasive mechanical ventilation [39], sparing patients from avoidable morbidity and lowering mortality from complications. Given the sensitivity of the model, it can effectively identify patients who are likely to be transferred to ICU within 24 h, reducing the chance of missing the patients in need of ICU care. Moreover, clinical implementation of the tool can increase the rates of early ICU transfers, which can potentially translate into reduced mortality and shorter lengths of ICU stay [40,41], with favorable consequences on other complications affecting patient outcomes, such as delirium and sleep disorders [42,43]. However, its positive predictive value and precision are limited, and it is not practical to perform labor-intensive interventions for all patients whom the model predicted are at high risk. Nonetheless, our model has clinical utility in the setting of a pandemic. The high negative predictive value suggests that those identified as unlikely to require critical care in the next 24 h may be considered for a lower level of monitoring.

## 5. Conclusions

Our RF-based tool can reliably be used for prioritizing COVID-19 patients not in the ICU but at risk for deterioration and requiring ICU transfer within 24 h. The model shows the importance of respiratory failure, shock, inflammation, and renal failure in the progression of COVID-19. Such a predictive tool may have wide implications and utility in clinical practice and hospital operations. Further refinement of the model will yield even higher precision while maintaining sensitivity. More studies are needed to identify other ways to improve patient outcomes by early identification of COVID-19 patients at risk of deterioration. Implementing machine learning models has the potential to build capacity within a hospital’s continuous learning and quality improvement environment.

## Figures and Tables

**Table 1:** Patient characteristics in the overall study cohort and test set.

	Overall (*n* = 1987)	Test (*n* = 612)
**Age Groups**		
18–45	643 (32.4%)	202 (33.0%)
45–65	638 (32.1%)	190 (31.0%)
65–80	491 (24.7%)	154 (25.2%)
>80	215 (10.8%)	66 (10.8%)
Gender		
Male	904 (45.5%)	283 (46.2%)
Female	1083 (54.5%)	329 (53.8%)
**Length of Stay**		
0–3 days	692 (34.8%)	216 (35.3%)
3–10 days	830 (41.8%)	256 (41.8%)
>10 days	332 (16.7%)	94 (15.4%)
Not discharged	133 (6.7%)	46 (7.5%)
**ICU care received**		
Yes	330 (16.6%)	103 (16.8%)
No	1657 (83.4%)	509 (83.2%)
**Geographic origin**		
Bronx	226 (11.4%)	68 (11.1%)
Brooklyn	330 (16.6%)	111 (18.1%)
Manhattan	833 (41.9%)	256 (41.8%)
Queens	339 (17.1%)	103 (16.8%)
Staten Island	27 (1.4%)	8 (1.3%)
Out of New York City	174 (8.8%)	48 (7.8%)
Out of NY State	57 (2.9%)	17 (2.8%)
Missing	1 (0.1%)	1 (0.2%)
**Race and Ethnicity**		
Non-Hispanic Black	331 (16.7%)	103 (16.8%)
Non-Hispanic White	546 (27.5%)	168 (27.5%)
Hispanic Black	45 (2.3%)	12 (2.0%)
Hispanic White	75 (3.8%)	23 (3.8%)
Asian	115 (5.8%)	35 (5.7%)
Others	739 (37.2%)	227 (37.1%)
Unspecified	136 (6.8%)	44 (7.2%)
**Smoking history**		
Current Smoker	105 (5.3%)	34 (5.6%)
Past smoker	431 (21.7%)	135 (22.1%)
Never smoked	1120 (56.4%)	339 (55.4%)
Unknown	324 (16.3%)	104 (17.0%)
Missing	7 (0.4%)	0 (0%)
**Hypertension**		
Yes	566 (28.5%)	162 (26.5%)
No	1414 (71.2%)	450 (73.5%)
Missing	7 (0.4%)	0 (0%)
**Diabetes**		
Yes	431 (21.7%)	140 (22.9%)
No	1549 (78.0%)	472 (77.1%)
Missing	7 (0.4%)	0 (0%)
**COPD and Asthma**		
Yes	170 (8.6%)	49 (8.0%)
No	1810 (91.1%)	563 (92.0%)
Missing	7 (0.4%)	0 (0%)
**Obesity**		
Yes	176 (8.9%)	53 (8.7%)
No	1804 (90.8%)	559 (91.3%)
Missing	7 (0.4%)	0 (0%)

**Table 2:** Predictive performance of the ICU prediction model in the test set.

Model	Total Feature Vectors in Test Cohort	% Prevalence of Positive Label *	Sensitivity (%)	Specificity (%)	Accuracy (%)	Precision (%)	Negative Predictive Value (%)	AUC-ROC (%)
Random Forest classifier	2812	3.7	72.8 (63.2–81.1)	76.3 (74.7–77.9)	76.2 (74.6–77.7)	10.5 (8.3–12.9)	98.7 (98.1–99.1)	79.9 (75.2–84.6)

* feature vectors labeled positive because ICU transfer occurred within 24 h of admission. AUC-ROC: area under receiver operating characteristic curve.

## Supplementary Materials

The following are available online at https://www.mdpi.com/2077-0383/9/6/1668/s1, Table S1: Hyperparameters used in the final model, Table S2: Variables included in the final model and their respective source.

## References

[1] World Health Organization WHO COVID-19 Dashboard. https://who.sprinklr.com/.

[2] Fauci A.S., Lane H.C., Redfield R.R. (2020). Covid-19—Navigating the Uncharted. N. Engl. J. Med..

[3] (2020). CDC COVID-19 Response Team. Severe Outcomes Among Patients with Coronavirus Disease 2019 (COVID-19)—United States, February 12–March 16, 2020. MMWR Morb. Mortal. Wkly. Rep..

[4] Myers L.C., Parodi S.M., Escobar G.J., Liu V.X. (2020). Characteristics of Hospitalized Adults With COVID-19 in an Integrated Health Care System in California. JAMA.

[5] Richardson S., Hirsch J.S., Narasimhan M., Crawford J.M., McGinn T., Davidson K.W., Cookingham J. (2020). Presenting Characteristics, Comorbidities, and Outcomes Among 5700 Patients Hospitalized With COVID-19 in the New York City Area. JAMA.

[6] Howell E. (2008). Active Bed Management by Hospitalists and Emergency Department Throughput. Ann. Intern. Med..

[7] Moriarty J.P., Schiebel N.E., Johnson M.G., Jensen J.B., Caples S.M., Morlan B.W., Huddleston J.M., Heubner M., Naessens J.M. (2014). Evaluating implementation of a rapid response team: Considering alternative outcome measures. Int. J. Qual. Health Care.

[8] Verity R., Okell L.C., Dorigatti I., Winskill P., Whittaker C., Imai N., Cori A., Fu H., Baguelin M., Dighe A. (2020). Estimates of the severity of coronavirus disease 2019: A model-based analysis. Lancet Infect. Dis..

[9] CDC COVID-19 Response Team (2020). Preliminary Estimates of the Prevalence of Selected Underlying Health Conditions Among Patients with Coronavirus Disease 2019—United States, February 12–March 28, 2020. MMWR Morb. Mortal. Wkly. Rep..

[10] American Hospital Association (2020). Fast Facts on U.S. Hospitals, 2020|AHA. 2018 AHA Annual Survey. https://www.aha.org/statistics/fast-facts-us-hospitals.

[11] Halpern N.A., Goldman D.A., Tan K.S., Pastores S.M. (2016). Trends in Critical Care Beds and Use Among Population Groups and Medicare and Medicaid Beneficiaries in the United States. Crit. Care Med..

[12] Halpern N.A., Tan K.S. (2020). United States Resource Availability for COVID-19. https://sccm.org/getattachment/Blog/March-2020/United-States-Resource-Availability-for-COVID-19/United-States-Resource-Availability-for-COVID-19.pdf.

[13] National Center for Immunization and Respiratory Diseases (NCIRD) (2020). Interim Clinical Guidance for Management of Patients with Confirmed Coronavirus Disease (COVID-19). https://www.cdc.gov/coronavirus/2019-ncov/hcp/clinical-guidance-management-patients.html.

[14] Tang N., Li D., Wang X., Sun Z. (2020). Abnormal coagulation parameters are associated with poor prognosis in patients with novel coronavirus pneumonia. J. Thromb. Haemost..

[15] Breiman L. (2001). Random Forrests. Mach. Learn..

[16] Basu S., Kumbier K., Brown J.B., Yu B. (2018). Iterative random forests to discover predictive and stable high-order interactions. Proc. Natl. Acad. Sci. USA.

[17] Batista G.E.A.P.A., Monard M.C. (2003). An analysis of four missing data treatment methods for supervised learning. Appl. Artif. Intell..

[18] Japkowicz N. The Class Imbalance Problem: Significance and Strategies. Proceedings of the 2000 International Conference on Artificial Intelligence.

[19] Kia A., Timsina P., Joshi H.N., Klang E., Gupta R.R., Freeman R.M., Reich D.L., Tomlinson M.S., Dudley J.T., Mazumdar M. (2020). MEWS++: Enhancing the Prediction of Clinical Deterioration in Admitted Patients through a Machine Learning Model. J. Clin. Med..

[20] (2018). The Apache Software Foundation. MLlib: Main Guide—Spark 2.3.0 Documentation. https://spark.apache.org/docs/2.3.0/mllib-ensembles.html.

[21] Nicastri E., Petrosillo N., Bartoli T.A., Lepore L., Mondi A., Palmieri F., Antinori A. (2020). National Institute for the Infectious Diseases “L. Spallanzani” IRCCS. Recommendations for COVID-19 Clinical Management. Infect. Dis. Rep..

[22] Zhou F., Yu T., Du R., Fan G., Liu Y., Liu Z., Xiang J., Wang Y., Song B., Guan L. (2020). Clinical course and risk factors for mortality of adult inpatients with COVID-19 in Wuhan, China: A retrospective cohort study. Lancet.

[23] Guo T., Fan Y., Chen M., Wu X., Zhang L., He T., Wang H., Wan J., Wang X., Lu Z. (2020). Cardiovascular Implications of Fatal Outcomes of Patients With Coronavirus Disease 2019 (COVID-19). JAMA Cardiol..

[24] Maxim L.D., Niebo R., Utell M.J. (2014). Screening tests: A review with examples. Inhal. Toxicol..

[25] R Core Team (2019). R: A Language and Environment for Statistical Computing.

[26] Grau J., Grosse I., Keilwagen J. (2015). PRROC: Computing and visualizing precision-recall and receiver operating characteristic curves in R. Bioinformatics.

[27] Robin X., Turck N., Hainard A., Tiberti N., Lisacek F., Sanchez J.C., Müller M. (2011). pROC: An open-source package for R and S+ to analyze and compare ROC curves. BMC Bioinform..

[28] Stevenson M., Nunes T., Sanchez J., Thornton R., Reiczigel J., Robison-Cox J., Sebastiani P. (2013). EpiR: An R Package for the Analysis of Epidemiological Data. http://www2.uaem.mx/r-mirror/web/packages/epiR/epiR.pdf.

[29] Cascella M., Rajnik M., Cuomo A., Dulebohn S.C., Di Napoli R. (2020). Features, Evaluation and Treatment Coronavirus (COVID-19).

[30] Qin C., Zhou L., Hu Z., Zhang S., Yang S., Tao Y., Xie C., Ma K., Shang K., Tian D.S. (2020). Dysregulation of Immune Response in Patients With Coronavirus 2019 (COVID-19) in Wuhan, China. Clin. Infect. Dis..

[31] Bhatraju P.K., Ghassemieh B.J., Nichols M., Kim R., Jerome K.R., Nalla A.K., Greninger A.L., Pipavath S., Wurfel M.M., Kritek P.A. (2020). Covid-19 in Critically Ill Patients in the Seattle Region—Case Series. N. Engl. J. Med..

[32] Matthay M.A., Aldrich J.M., Gotts J.E. (2020). Treatment for severe acute respiratory distress syndrome from COVID-19. Lancet Respir. Med..

[33] Wang L. (2020). C-reactive protein levels in the early stage of COVID-19. Méd. Mal. Infect..

[34] Kyriacos U., Jelsma J., Jordan S. (2011). Monitoring vital signs using early warning scoring systems: A review of the literature. J. Nurs. Manag..

[35] Aird W.C. (2003). The Hematologic System as a Marker of Organ Dysfunction in Sepsis. Mayo Clin. Proc..

[36] Lippi G., South A.M., Henry B.M. (2020). Electrolyte Imbalances in Patients with Severe Coronavirus Disease 2019 (COVID-19). Ann. Clin. Biochem..

[37] Goyal P., Choi J.J., Pinheiro L.C., Schenck E.J., Chen R., Jabri A., Satlin M.J., Nahid M., Nahid J.B., Hoffman K.L. (2020). Clinical Characteristics of Covid-19 in New York City. N. Engl. J. Med..

[38] Lippi G., Plebani M. (2020). Laboratory abnormalities in patients with COVID-2019 infection. Clin. Chem. Lab. Med..

[39] Sun Q., Qiu H., Huang M., Yang Y. (2020). Lower mortality of COVID-19 by early recognition and intervention: Experience from Jiangsu Province. Ann. Intensive Care.

[40] Churpek M.M., Wendlandt B., Zadravecz F.J., Adhikari R., Winslow C., Edelson D.P. (2016). Association between intensive care unit transfer delay and hospital mortality: A multicenter investigation. J. Hosp. Med..

[41] Hu W., Chan C.W., Zubizarreta J.R., Escobar G.J. (2018). An examination of early transfers to the ICU based on a physiologic risk score. Manuf. Serv. Oper. Manag..

[42] Medrzycka-Dabrowska W., Lewandowska K., Kwiecień-Jagus K., Czyz-Szypenbajl K. (2018). Sleep deprivation in Intensive Care Unit-systematic review. Open Med..

[43] Kotfis K., Williams Roberson S., Wilson J.E., Dabrowski W., Pun B.T., Ely E.W. (2020). COVID-19: ICU delirium management during SARS-CoV-2 pandemic. Crit. Care.

