# Supplementary material for: Using Machine Learning to Predict ICU Transfer in Hospitalized COVID-19 Patients
Source: J Clin Med. 2020 Jun 1;9(6):1668. doi: 10.3390/jcm9061668 (PMC7356638; doi:10.3390/jcm9061668)
Supplement: Supplementary file 1 [file jcm-09-01668-s001.pdf]

Supplementary Table 1. Hyperparameters used in the final model

**Hyperparameter**	**Final value**
Number of trees to train	100
Maximum depth of the tree	4
Maximum number of bins for discretizing continuous features	15
Number of features to consider for splits at each tree node	One third

Supplementary Table 2. Variables included in the final model and their respective source

**Variable names**	**Source**
Age	Patient
Gender	Patient
Pulse Rate	Vitals Flowsheet
Respiratory Rate	Vitals Flowsheet
Oral Temperature	Vitals Flowsheet
Percutaneous oxygen saturation (pulse oximetry)	Vitals Flowsheet
Systolic Blood Pressure	Vitals Flowsheet
Diastolic Blood Pressure	Vitals Flowsheet
Red blood cell count	Laboratory
Lymphocyte count	Laboratory
Platelet count	Laboratory
White blood cell count	Laboratory
Serum Sodium	Laboratory
Serum Potassium	Laboratory
Serum Chloride	Laboratory
Serum Calcium	Laboratory
Blood urea nitrogen	Laboratory
Serum creatinine	Laboratory
Hemoglobin	Laboratory
Serum albumin	Laboratory
Serum total protein	Laboratory
C reactive protein	Laboratory
Activated partial thromboplastin time	Laboratory
Anion gap	Laboratory
P axis	Electrocardiogram
PR interval	Electrocardiogram
QRS Duration	Electrocardiogram
R Axis	Electrocardiogram
T Axis	Electrocardiogram
Atrial Rate	Electrocardiogram

Ventricular Rate	Electrocardiogram
